# Real-time tracking of ER turnover during ERLAD by a rhenium complex via lifetime imaging

**DOI:** 10.1093/nsr/nwab194

**Published:** 2021-10-28

**Authors:** Liang Hao, Yu-Yi Ling, Zhi-Xin Huang, Zheng-Yin Pan, Cai-Ping Tan, Zong-Wan Mao

**Affiliations:** MOE Key Laboratory of Bioinorganic and Synthetic Chemistry, School of Chemistry, State Key Laboratory of Oncology in South China, Sun Yat-Sen University, Guangzhou 510275, China; MOE Key Laboratory of Bioinorganic and Synthetic Chemistry, School of Chemistry, State Key Laboratory of Oncology in South China, Sun Yat-Sen University, Guangzhou 510275, China; MOE Key Laboratory of Bioinorganic and Synthetic Chemistry, School of Chemistry, State Key Laboratory of Oncology in South China, Sun Yat-Sen University, Guangzhou 510275, China; MOE Key Laboratory of Bioinorganic and Synthetic Chemistry, School of Chemistry, State Key Laboratory of Oncology in South China, Sun Yat-Sen University, Guangzhou 510275, China; MOE Key Laboratory of Bioinorganic and Synthetic Chemistry, School of Chemistry, State Key Laboratory of Oncology in South China, Sun Yat-Sen University, Guangzhou 510275, China; MOE Key Laboratory of Bioinorganic and Synthetic Chemistry, School of Chemistry, State Key Laboratory of Oncology in South China, Sun Yat-Sen University, Guangzhou 510275, China

**Keywords:** rhenium, ER-phagy, viscosity, TPFLIM

## Abstract

Endoplasmic reticulum (ER) degradation by autophagy (ER-phagy) is a recently revealed selective autophagy pathway that plays important roles in organelle turnover and protein degradation, but the biological functions of ER-phagy are largely unknown. Here, we present an ER-targeting Re(I) tricarbonyl complex (Re-ERLAD) that can accumulate in the ER, induce ER-to-lysosome-associated degradation (ERLAD) upon visible light irradiation, and label ER buds and track their morphological alterations during ER-phagy. The emission of Re-ERLAD is sensitive to viscosity, which is a key parameter reflecting the amount of unfolded protein in the ER. Quantitative detection using two-photon fluorescence lifetime imaging microscopy shows that ER viscosity initially increases and then decreases during ERLAD, which reveals that ERLAD is a pathway for alleviating ER stress caused by unfolded proteins. In conclusion, our work presents the first specific photoinducer and tracker of ERLAD, which can be used in studying the regulatory mechanism and function of this process.

## Teaser

The first specific inducer and tracker of ERLAD was reported and used to track the dynamic change of ER buds viscosity during ERLAD.

## INTRODUCTION

The endoplasmic reticulum (ER) plays important roles in protein and lipid synthesis, ion homeostasis and organelle communication [1]. To protect cells from both endogenous and exogenous threats, e.g. unfolded proteins [2] or reactive oxygen species (ROS) [3], the ER has developed complicated self-modulation mechanisms [4–6]. Depending on the receptors involved and substrates degraded, at least three subtypes of ER degradation by autophagy (ER-phagy) have been reported in mammalian cells: macro ER-phagy, ER-to-lysosome-associated degradation (ERLAD) by micro ER-phagy and ER-phagy-related ERLAD [7]. Compared with ER-associated degradation (ERAD) [8], ERLAD provides a proteasome-free degradation pathway for protein quality control, which is crucial for ER turnover and cellular homeostasis [9–11]. However, very limited tools, including specific inducers and imaging agents, are currently available for the investigation of the regulatory mechanisms and functions of ER-phagy.

Viscosity is an important micro-environmental factor reflecting the functionalities of organelles, including mitochondria, lysosomes and the ER. Viscosity is closely related to intracellular diffusion, molecular interactions and membrane fluidity, which are crucial for biological interactions and biochemical reactions. Intracellular viscosity can be reflected by molecular rotors, whose emission is quenched by intramolecular rotation and recovers upon an increase in environmental viscosity [12–14]. The lifetime of a fluorophore does not depend on its concentration; however, it is sensitive to its environment [14]. Using fluorescence lifetime imaging microscopy (FLIM), environmental parameters, e.g. viscosity and pH, can be measured quantitatively with high accuracy [15–17]. Via exploitation of their intrinsic subcellular localization properties or modification with targeting groups, these molecular rotors can be used to measure the viscosity of subcellular organelles, e.g. mitochondria [16], lysosomes [15] and the ER [12].

Theranostic small molecules that can induce and be used to monitor a specific biological process have many advantages, including simplified operational procedures and minimized cross-interference of reagents [18–29]. Light-triggered theranostic reagents that can reveal biological phenomena in a controllable manner are particularly attractive [30]. Transition metal complexes are widely investigated as antitumor agents [31–34] or biological probes [35–37]. Among these complexes, rhenium tricarbonyl complexes show significant anticancer activity and fluorescent properties [38–45], which makes them appropriate candidates as theranostic agents.

**Figure 1.**
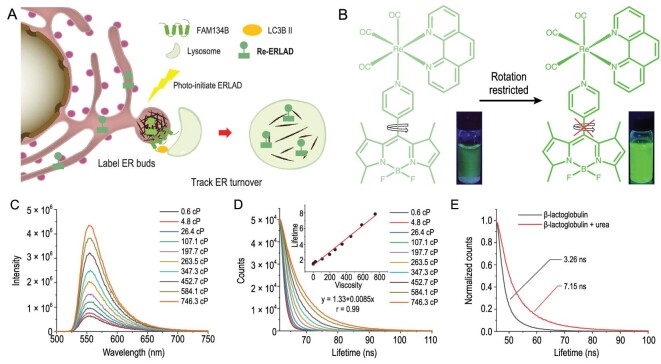
(A) Illustration of the mechanisms of induction and tracking of ERLAD by **Re-ERLAD**. (B) Chemical structure of **Re-ERLAD** and mechanism of its response to viscosity. (C) Emission intensity and (D) lifetime spectra of **Re-ERLAD** (10 μM) in the methanol-glycerol system representing different viscosities. *λ*_ex _= 405 nm. (E) Lifetime of **Re-ERLAD** (10 μM) in aqueous solutions of folded/unfolded β-lactoglobulin (1 mM). β-lactoglobulin is unfolded with urea (6 M) for 24 h. *λ*_ex _= 405 nm. Centipoise (cP) is a common unit for dynamic viscosity; 1 cP = 10^–3^ Pa·s.

To date, there have been few reports on the monitoring of dynamic turnover behavior at the subcellular level, which is particularly important for the maintenance of cell homeostasis. In this work, we attached the molecular rotor BODIPY [46,47] to the organometallic rhenium tricarbonyl moiety to produce an ER-targeting phototheranostic agent (**Re-ERLAD**) with a viscosity-sensitive emission property and lifetime (Fig. 1A). The attachment of the rhenium moiety to BODIPY alters its cellular localization and significantly increases its capability to generate singlet oxygen (^1^O_2_) upon irradiation due to the heavy atom effect. **Re-ERLAD** can induce ERLAD upon photoinitiation, specifically imaging ER buds (a special microstructure formed during ERLAD) and quantitatively tracking the viscosity parameters in ER buds during ER-phagy via two-photon fluorescence lifetime imaging microscopy (TPFLIM). The light-activated property makes **Re-ERLAD** more controllable and widely applicable than previously reported theranostic agents [18,19], since it can be used as a simple probe to measure the changes in ER viscosity initiated by other stimuli. Overall, we present here the first report on a specific photoinducer and viscosity tracker of ERLAD, which shows the dynamic changes in micro-environments during ER turnover.

## RESULTS AND DISCUSSION

### Synthesis and characterization of the Re-ERLAD

The ligand **py-BODIPY** was synthesized by a coupling reaction of 2,4-dimethylpyrrole with 4-pyridinecarboxaldehyde in CH_2_Cl_2_ (Scheme S1). **Re-ERLAD** was synthesized by reacting the precursor [Re(CO)_3_(phen)Cl] (phen = 1,10-phenanthroline) with **py-BODIPY**, and purified by silica chromatography. **Py-BODIPY** and **Re-ERLAD** were characterized by ESI-MS, ^1^H NMR spectroscopy and HPLC (Figs S1–S6).

In CH_2_Cl_2_, CH_3_CN and PBS, **py-BODIPY** shows an absorption band at 425–525 nm, while **Re-ERLAD** shows an intense absorption band at 440–550 nm assigned to the BODIPY group and less intense absorption bands at 350–425 nm assigned to a mixture of spin-allowed and spin-forbidden metal-to-ligand charge transfer transitions (^1^MLCT/^3^MLCT; Fig. S7). Upon excitation at 405 nm, **py-BODIPY** exhibits strong luminescence with a maximum at *ca.* 520 nm, and **Re-ERLAD** shows a broader emission band centered at *ca.* 530 nm (Fig. S8). The quantum yield of **Re-ERLAD** is lower than that of **py-BODIPY**, which may be attributed to the energy transfer from the BODIPY ligand to the metal center [48]. The photostability of **Re-ERLAD** under physiological conditions was also verified by HPLC (Fig. S9). **Re-ERLAD** remains stable after light irradiation, which indicates that **Re-ERLAD** is suitable for long-term imaging in living cells.

### Viscosity response of Re-ERLAD

The fluorescence response of **Re-ERLAD** to environmental viscosity was measured in a mixed methanol-glycerol system representing different viscosities. **Re-ERLAD** exhibits *ca.* 8-fold emission enhancement in high-viscosity media compared with low-viscosity media (Fig. 1C). The emission recovery that can be recognized by the naked eye is ascribed to the restricted rotation of **py-BODIPY**. In addition, the fluorescence lifetime of **Re-ERLAD** increases from 1.5 ns to 7.9 ns and shows a linear correlation with the viscosity parameters (Fig. 1D). Moreover, the emission intensity and lifetime of **Re-ERLAD** show negligible responses to polarity, common cations, glutathione and human serum albumin, which indicates the specificity of the fluorescence response of **Re-ERLAD** to viscosity (Figs S10 and S11).

Disrupted protein folding and accumulation of unfolded proteins can cause oxidative damage and ER stress [49,50]. The impact of unfolded proteins on the solution viscosity was investigated by using β-lactoglobulin as a model protein and urea as the unfolding agent. As expected, the mobility of the protein solution significantly decreased after treatment with urea for 24 h, and the lifetime of **Re-ERLAD** was obviously enhanced in the solution containing unfolded proteins (Fig. 1E). The results indicate that the changes in environmental viscosity are correlated with the degree of protein unfolding, which can be reflected by the emission intensity and lifetime of **Re-ERLAD**.

### Cytotoxicity and cellular localization of Re-ERLAD

The *in vitro* cytotoxicities of **py-BODIPY** and **Re-ERLAD** were evaluated in A549 (human lung adenocarcinoma) cells after 48 h of incubation (Fig. S12). **Re-ERLAD** shows low cytotoxicity in the dark, while it exhibits obviously increased cytotoxicity after irradiation with a 450 nm laser for 15 min. In contrast, **py-BODIPY** is non-cytotoxic both in the dark and under irradiation. Under 450 nm light irradiation, the quantum yields of ^1^O_2_ photosensitization for **py-BODIPY** and **Re-ERLAD** are 0.07 and 0.28, respectively. The higher photosensitization capability of **Re-ERLAD** can be ascribed to the heavy atom effect of the metal center [51]. Compared to the control group, the emission of the ROS probe significantly increased by ∼17-fold in A549 cells treated with **Re-ERLAD** and irradiated with a 450 nm laser for 15 min (Fig. S13).

Many organometallic rhenium tricarbonyl complexes have been reported to accumulate in mitochondria [33]; however, colocalization studies have revealed that the positively charged **Re-ERLAD** localizes in the ER. The colocalization coefficient of **Re-ERLAD** with the ER (0.97; Fig. S14A) was much higher than that of **Re-ERLAD** with mitochondria (0.37; Fig. S14B). The neutral hydrophobic molecule **py-BODIPY** shows a high colocalization coefficient (0.89) with the lipid droplet-specific fluorescent dye (Fig. S14C). These results suggest that the increased photocytotoxicity of **Re-ERLAD** compared with **py-BODIPY** is attributed to its ability to produce large amounts of cellular ROS and may also be associated with its subcellular localization properties.

### Re-ERLAD initiates ERLAD

**Figure 2.**
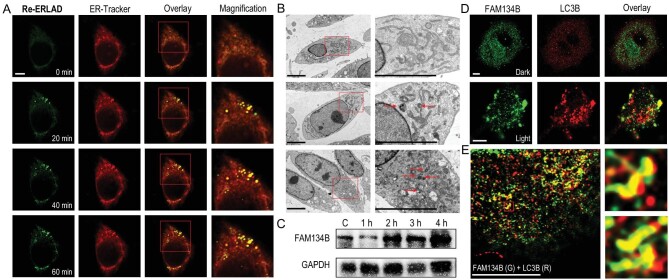
(A) ER fragments budding from reticular structures. A549 cells were incubated with **Re-ERLAD** (1 μM, 1 h) and ER-Tracker Red (1 μM, 30 min) before irradiation with a 450 nm laser for 15 min. **Re-ERLAD**: *λ*_ex _= 405 nm; *λ*_em _= 570 ± 20 nm. ER-Tracker Red: *λ*_ex _= 561 nm; *λ*_em _= 610 ± 20 nm. Scale bar: 5 μm. (B) TEM observation of A549 cells incubated with **Re-ERLAD** (1 μM, 1 h) and irradiated with a 450 nm laser for 0 (top), 10 (middle) or 20 (bottom) min and further incubated for 4 h. ER buds and fragments are labeled with arrowheads. Scale bars: 5 μm. (C) Western blot of FAM134B. A549 cells were incubated with **Re-ERLAD** (1 μM, 1 h) before irradiation with a 450 nm laser for 15 min and further incubation for 1–4 h. (D) Interaction of FAM134B with LC3B captured by confocal microscopy. A549 cells were incubated with **Re-ERLAD** (1 μM, 1 h) before irradiation at 450 nm for 15 min and further incubation for 1 h. FAM134B and LC3B were stained by immunofluorescence. FAM134B: *λ*_ex _= 561 nm; *λ*_em _= 610 ± 20 nm. LC3B: *λ*_ex _= 633 nm; *λ*_em _= 660 ± 20 nm. Scale bars: 5 μm. (E) Super-resolution images of FAM134B interacting with LC3B. A549 cells were incubated with **Re-ERLAD** (1 μM, 1 h) before irradiation at 450 nm for 15 min and further incubation for 1 h. G = green; R = red. Scale bar: 5 μm.

**Re-ERLAD** colocalizes with ER-Tracker and shows a weaker emission intensity in the dark. After irradiation with a 450 nm laser for 15 min, however, highly emissive punctate areas that colocalized with ER-Tracker were observed (Fig. 2A). These structures show minimal colocalization with fluorescent probes that stain punctate subcellular organelles, including lipid droplets and lysosomes (Fig. S15). We speculate that these punctate areas are likely to be ER buds grown from the reticular structure of the ER [11], and the strong emission may be attributed to the accumulation of unfolded proteins caused by light-induced oxidative damage.

To confirm what these highly emissive structures are, transmission electron microscopy (TEM) was used to observe the morphological alterations in the ultrastructure of A549 cells. Compared with the control group, ER fragments and vesicles with monolayer structures and contents inside were found in cells treated with **Re-ERLAD** in the presence of light (Fig. 2B). In addition, ER buds with a monolayer membrane budding from reticular structures were also observed. These structural changes are consistent with the phenomenon described for ERLAD, which forms monolayer vesicles rather than bilayer autophagosomes to fuse with lysosomes [7]. Time-dependent upregulation of the key ER-phagy receptor FAM134B, which is involved in ERLAD by generating high membrane curvature [52], was further confirmed by western blotting (Fig. 2C). Similar results were also obtained for the positive controls rapamycin and tunicamycin, which are reported to induce ER stress and ER-phagy in the literature (Fig. S16) [9]. In addition, the interaction between FAM134B and LC3B, two key proteins regulating the process of ERLAD, was confirmed with immunofluorescence double staining (Fig. 2D), and the overlap coefficient increased from 0.01 (dark) to 0.31 (light). The interactions of FAM134B and LC3B were also detected by Airyscan super-resolution imaging techniques (Fig. 2E). Collectively, these results indicate that **Re-ERLAD** can specifically initiate the ERLAD process upon irradiation.

**Figure 3.**
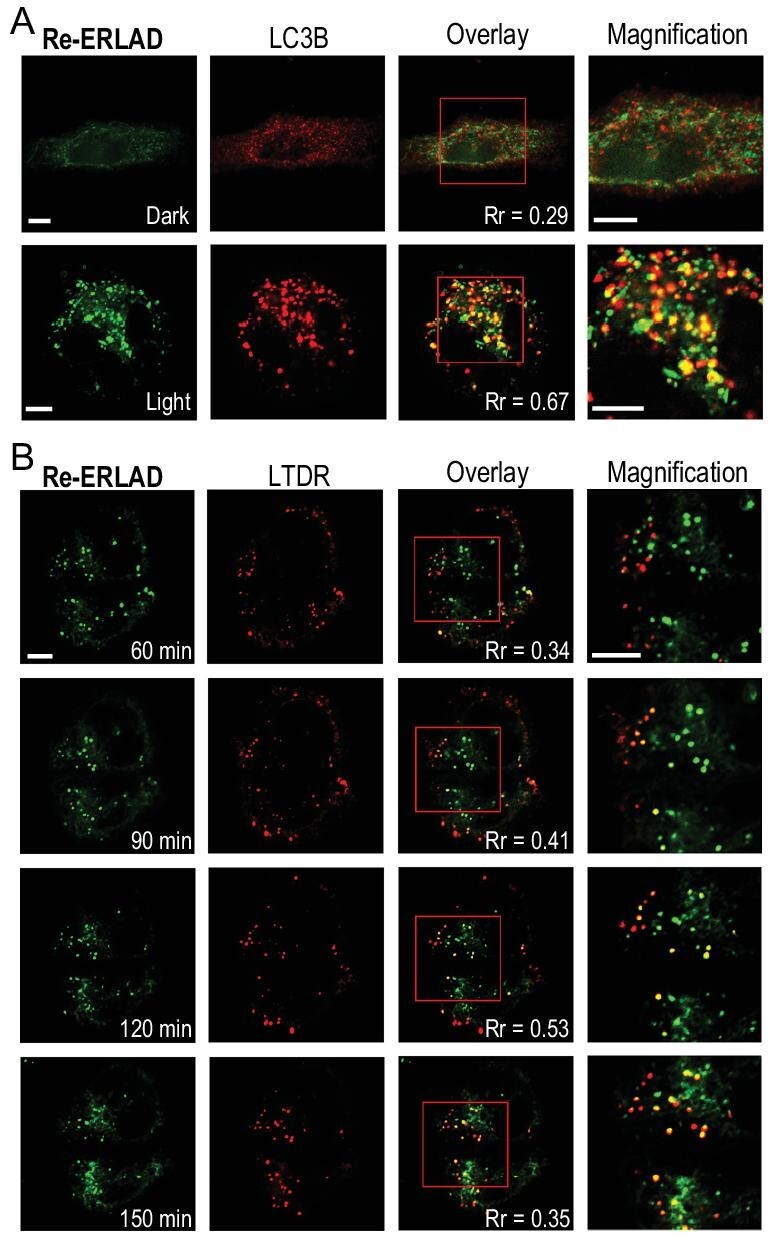
Tracking of key events of ER buds during ERLAD by **Re-ERLAD**. (A) Colocalization of ER buds with LC3B. Cells transfected with RFP-LC3B were incubated with **Re-ERLAD** (1 μM, 1 h) before irradiation at 450 nm for 15 min and further incubation for 1 h. Scale bars: 5 μm. (B) Fusion of ER buds with lysosomes. Cells were incubated with **Re-ERLAD** (1 μM, 1 h) and ER-Tracker Red (1 μM, 30 min) before irradiation at 450 nm for 15 min. *λ*_ex_ = 405 nm; *λ*_em_ = 570 ± 20 nm. LC3B: *λ*_ex_ = 561 nm; *λ*_em_ = 610 ± 20 nm. LTDR: 633 nm; *λ*_em_ = 660 ± 20 nm (LC3B). Rr: Pearson correlation coefficient. Scale bars: 5 μm.

Then, we used **Re-ERLAD** to track ER-associated morphological events during **ERLAD**. Before irradiation, the emission of **Re-ERLAD** was very weak, and there was no obvious colocalization between **Re-ERLAD** and LC3B. After irradiation, the punctate emission of **Re-ERLAD** was greatly enhanced, and colocalization of **Re-ERLAD** with punctate LC3B was detected, which showed that ER buds were formed and began to recruit LC3B (Fig. 3A). At the same time, with prolonged incubation time, the punctate emission of **Re-ERLAD** gradually fuses with Lyso-Tracker Deep Red (LTDR) and disappears, indicating that ER buds are gradually engulfed by lysosomes (Fig. 3B). The Pearson correlation coefficients increase gradually from 60 min to 120 min but decrease at 150 min. The reason might be that the signal intensity of **Re-ERLAD** is weakened along with the degradation of ER buds in lysosomes at 150 min. These results show that **Re-ERLAD** can track the morphological alterations in ER buds during ERLAD [53].

### Real-time tracking of ERLAD with TPFLIM

As **Re-ERLAD** can induce ERLAD in the presence of light, specifically label ER buds and possess viscosity-dependent emission properties, we then used it as a theranostic probe to monitor the changes in the viscosity of ER buds during light-initiated ERLAD via TPFLIM. The lifetimes acquired from TPFLIM were plugged into the correlation curve in Fig. 1D to give the viscosity values. Considering the complicated cellular environment, the viscosity values may not be very accurate. Therefore, the trend of changes in viscosity is more meaningful and will give us more information than the specific values.

**Figure 4.**
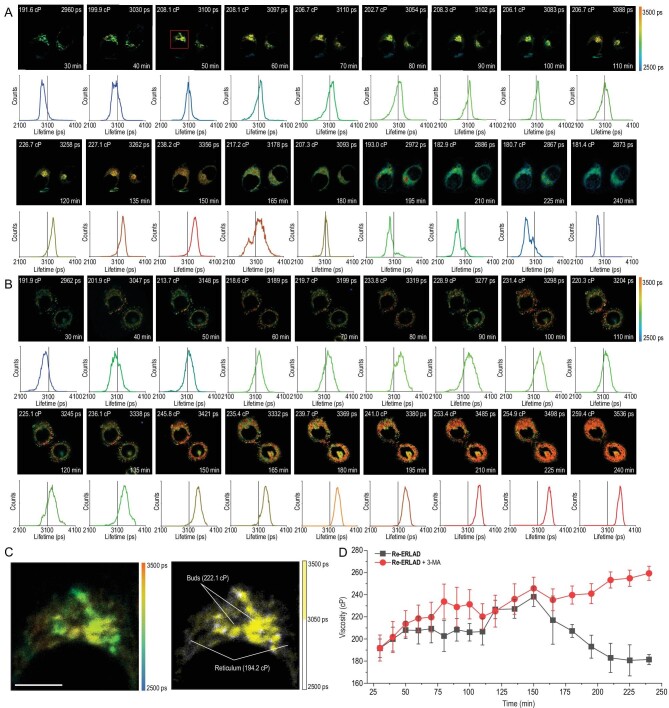
Real-time tracking of ER bud viscosity in (A) A549 cells and (B) 3-MA (5 mM)-pretreated A549 cells via TPFLIM. Cells were treated with **Re-ERLAD** for 1 h and irradiated with 450 nm for 15 min. *λ*_ex _= 810 nm; *λ*_em _= 550∼595 nm. (C) Time-gated TPFLIM image of ER buds separated from the reticular structure. The image was magnified from the area in the red frame in (A). (D) Trend chart showing the calculated viscosity during the process. Cells were irradiated at 450 nm for 15 min and were then visualized by TPFLIM. Scale bar: 5 μm. The lifetime value was given by Becker & Hickl's SPCImage software.

To study the dynamic change in the viscosity of ER buds during ERLAD, a long period of real-time monitoring was carried out. Interestingly, we found that the changes in viscosity reflecting the degree of unfolded protein aggregation during ERLAD can be divided into two distinct stages. At the earlier stage, a time-dependent increase in viscosity is observed in A549 cells treated with **Re-ERLAD** upon light irradiation (Fig. 4A), during which ER buds with higher viscosity are separated from the reticular structure (Fig. 4C). The growth is sustained for 2.5 h until the viscosity increases to *ca.* 240 cP from *ca.* 190 cP. The increase in viscosity indicates the gradual aggregation of unfolded proteins in ER buds, which may serve as a signal to activate ERLAD [10,53]. At the later stage (2.5∼4 h), the exorbitant viscosity gradually decreases to *ca.* 180 cP. According to the results from the colocalization experiment, the decrease in viscosity implies the lysosome-mediated degradation of ER buds, which serves as a cellular turnover process to relieve ER stress.

However, when 3-methyladenine (3-MA; a specific inhibitor of autophagy that blocks blocking autophagosome formation) is introduced to the process, the turnover process reflected by the viscosity is reversed (Fig. 4B and D). Instead, the increase in ER viscosity is prolonged, increasing to *ca.* 260 cP at 4 h, accompanied by obvious cell shrinkage and cellular vacuolation. Because 3-MA reverses the ER turnover process by inhibiting ER-phagy, the survival rates of **Re-ERLAD**-treated cells decrease in the presence of light (Fig. S17), which indicates that ERLAD is a cytoprotective mechanism against **Re-ERLAD**-induced cell death. Calreticulin is correlated with immunogenic cell death, which could be induced by ER damage [54,55]. Consistent with the enhancing effects of 3-MA on the photocytotoxic effects of **Re-ERLAD**, a higher rate of calreticulin expression is observed in 3-MA-treated cells (Fig. S18). These results show that **Re-ERLAD** can induce and be used to monitor the dynamic changes in the viscosity of ER buds during photoinitiated ERLAD.

## CONCLUSION

Overall, we developed the first small molecule-based specific ERLAD inducer, **Re-ERLAD,** which has viscosity-sensitive emission properties and can specifically image ER buds. **Re-ERLAD** can photoinitiate ERLAD, a specific form of ER-phagy. **Re-ERLAD** can also be used to monitor morphological alterations in the ER during ERLAD, including accumulation of unfolded proteins in ER buds, recruitment of LC3B proteins and subsequent fusion with lysosomes for degradation. Moreover, **Re-ERLAD** can be used to quantitatively monitor the dynamic changes in the viscosity of ER buds during ERLAD via TPFLIM, revealing that the micro-environmental turnover of ER is cytoprotective. In summary, we present the first example of the dynamic turnover process of subcellular organelles by quantitatively measuring their micro-environmental parameters, an approach that may be used in ER monitoring or screening of compounds/methods for intervention with ERLAD.

## Supplementary Material

nwab194_Supplemental_File
