# Supplementary material for: Real-time tracking of ER turnover during ERLAD by a rhenium complex via lifetime imaging
Source: Natl Sci Rev. 2021 Oct 28;9(7):nwab194. doi: 10.1093/nsr/nwab194 (PMC9362766; doi:10.1093/nsr/nwab194)
Supplement: nwab194_Supplemental_File [file nwab194_supplemental_file.docx]

**Supplementary Information**

**Real-TimeTracking of ER Turnover during ERLAD by a Rhenium Complex via Lifetime Imaging**

Liang Hao, Yu-Yi Ling, Zhi-Xin Huang, Zheng-Yin Pan, Cai-Ping Tan,* and Zong-Wan Mao*

MOE Key Laboratory of Bioinorganic and Synthetic Chemistry, School of Chemistry, State Key Laboratory of Oncology in South China, Sun Yat-Sen University, Guangzhou 510275, P. R. China

**Table of Contents**

**Methods** **S3**

**Supplementary experimental section** **S4**

**Supporting Scheme, Figures and Tables** **S10**

**References** **S19**

**Methods**

**Materials**

2,4-dimethylpyrrole (J&K Chemical, China), 4-pyridinecarboxaldehyde (J&K Chemical, China), BF_3_OEt_2_ (J&K Chemical, China), pentacarbonylchlororhenium (J&K Chemical, China), 1,10-Phenanthroline (J&K Chemical, China), 2,3-dicyano-5,6-dichlorobenzoquinone (J&K Chemical, China), triethylamine (J&K Chemical, China), dimethyl sulfoxide (DMSO, Sigma Aldrich, USA), glycerol (Gly, J&K Chemical, China), [Ru(bpy)_3_]Cl_2_ (bpy = 2,2-Bispyridine, Sigma Aldrich, USA), trypsin (Hyclone Laboratoreis Inc, USA), (9,10-Anthracenediyl-bis(methylene)-dimalonic acid (ABDA, J&K Chemical, China) Roswell Park Memorial Institute Medium (RPMI1640 Medium, Hyclone Laboratoreis Inc, USA), phosphate buffered saline (PBS, Sigma Aldrich, USA), fetal bovine serum (FBS, Hyclone Laboratoreis Inc), 3-(4,5-dimethyl-2-thiazolyl)-2,5-diphenyl-2-H-tetrazolium bromide (MTT, J&K Chemical, China), LysoTracker® Deep Red (LTDR, Life Technologies, USA), ER-Tracker Red (ERTR, Beyotime, China), LipidSpot 610 (Biotium, USA), Cell ROX™ Deep Red Reagent (ThermoFisher, USA), Mouse monoclonal [GT3612] to LC3B (Abcam, USA), Rabbit monoclonal [EPR10608(B)] to FAM134B (Abcam, USA), Rabbit monoclonal [EPR3924] to Calreticulin (Abcam, USA), DyeLight 561-conjugated anti-rabbit (Abcam, USA), DyeLight 649-conjugated anti-mouse (Abcam, USA), Phenylmethylsulfonyl fluoride (PMSF, J&K Chemical, China), Cell lysis buffer (Beyotime, China), QuickBlock™ (Beyotime, China), DAB Horseradish Peroxidase Color Development Kit (Beyotime, China), glutaraldehyde (Sigma Aldrich, China) were used as received. Other materials and chemicals were purchased from the commercial sources. All the tested compounds were dissolved in DMSO as mother liquor before diluted into the experimental concentration with 1% (v/v) DMSO in the solvents.

**Instrumentation**

^1^H NMR spectra were recorded on a Bruker Avance 400 spectrometer (Germany). Shifts were referenced relative to the internal solvent signals. ESI-MS were recorded on a Thermo Finnigan LCQ DECA XP spectrometer (USA). The quoted m/z values represented the major peaks in the isotopic distribution. Microanalysis (C, H, and N) was carried out using an Elemental Vario EL CHNS analyzer (Germany). HPLC spectra were carried out with a Hewlett Packard High Performance Liquid Chromatograph (USA). UV/Vis spectra were recorded on a Varian Cary 300 spectrophotometer (USA). Emission measurements were conducted on an FLS 920 combined fluorescence lifetime and steady state spectrometer (Japan). Quantum yields of luminescence at room temperature were calculated according to literature procedures using [Ru(bpy)_3_]Cl_2_ as the reference.^[1-3]^ Confocal and TPFLIM images were recorded on a Carl Zeiss LSM 810 laser scanning confocal microscope (Germany). The cofocal microscope was combined with a Becker & Hickl (BH) time-correlated single photon counting (TCSPC) system. The PLIM data were analyzed using the SPCImage software available on www.becker-hickl.com (Becker & Hickl GmbH, the bh TCSPC Handbook sixth Edition). Tecan Infinite M200 Pro microplate reader (Switzerland) was used in MTT assay. FluorChem M (Protein Simple, USA) was used for chemiluminescence detection in western blot.

**Supplementary experimental section**

**Synthetic protocols and characterizations**

**Py-BODIPY**: The substituted two equivalents 2,4-dimethylpyrrole (3.95 g, 34 mmol) and 4-pyridinecarboxaldehyde (1.82 g, 17 mmol) were dissolved under N_2_ atmosphere in dichloromethane (300 mL). Several drops of trifluoroacetic acid were added and the mixture was stirred for 4 days at room temperature. After 4 days, the 2,3-dicyano-5,6-dichlorobenzoquinone (DDQ, 3.867 g, 17 mmol) was added into the reaction and stirred for 1 h. Subsequently, the triethylamine (TEA, 25 ml, 180 mmol) and BF_3_OEt_2_ (30 ml, 240 mmol) were added into the reaction in ice-cold condition and stirred over night at room temperature. The mixture was washed with brine (100 mL). The organic layer was dried with anhydrous Na_2_SO_4_ and spin dried in vacuum. The residue was purified by silica chromatography using CH_2_Cl_2_/EtOAc (50:1, V/V) to afford the red solid. Yield: 1.105 g (20%). ^1^H NMR (400 MHz, Chloroform-*d*) δ 8.81 (d, *J* = 4.7 Hz, 2H), 7.36 (d, *J* = 4.7 Hz, 2H), 6.04 (s, 2H), 2.59 (s, 6H), 1.43 (s, 6H). ESI-MS (CH3OH): m/z calcd (%) for; found:. Purity = 100% (by HPLC)

**Re-ERLAD**: **[**Re(CO)_5_Cl] (722 mg, 2 mmol) and 1,10-Phenanthroline (phen, 360 mg, 2 mmol) were dissolved in toluene and heated under reflux in an N_2_ atmosphere for 6 h and filtered to obtain the yellow solid [Re(CO)_3_(phen)Cl]. After that, a solution of AgOTf (256.9 mg, 1 mmol) and [Re(CO)_3_(phen)Cl] (477 mg, 0.98 mmol) in THF was heated under reflux in an N_2_ atmosphere for 1 h and filtered. Then, **py-BODIPY** (325 mg, 1 mmol) was added to the solution and heated under reflux for 24 h. The solvent is removed on a rotary evaporator and the solid was purified by silica chromatography using CH_2_Cl_2_/EtOH (9:1, v/v) to obtain a red solid. Yield: 0.673 g (68%). ^1^H NMR (400 MHz, Chloroform-d) *δ* 9.67 (dd, *J* = 5.1, 1.2 Hz, 2H), 8.84 (dd, *J* = 8.3, 1.2 Hz, 2H), 8.47 (d, *J* = 6.5 Hz, 2H), 8.25 (dd, *J* = 8.3, 5.1 Hz, 2H), 8.20 (s, 2H), 7.26 (d, *J* = 6.5 Hz, 2H), 5.89 (s, 2H), 2.48 (s, 6H), 0.79 (s, 6H). ESI-MS (CH_3_OH): *m/z* calcd (%) for [M−PF_6_]^+^, 776.17; found: 776.2. Elemental analysis calcd (%) for C_33_H_26_BF_8_N_5_O_3_Pre·3H_2_O: C, 40.67; H, 3.31; N, 7.19; found: C, 40.85; H, 3.34; N, 7.12. Purity > 95% (by HPLC).

**Photophysical properties**

The UV-Vis spectra, emission quantum yields and lifetimes of **Re-ERLAD** and **py-BODIPY** in PBS, CH_3_CN and CH_2_Cl_2_ at 298K were obtained on a Varian Cary 300 spectrophotometer (USA) and an FLS 920 combined fluorescence lifetime and steady spectrometer (Japan). The fluorescent quantum yields were calculated using [Ru(bpy)_3_]Cl_2_ as standards.^[1-3]^ The resulting data were processed with Origin Pro v8.0. All media contain minimum DMSO (1% v/v) for better solvency.

**Viscosity-responsive emission properties**

The emission properties of **Re-ERLAD** in mixed solvents containing methanol and glycerol representing different viscosity (percentage of glycerol: 0%, 10%, 20%, 30%, 40%, 50%, 60%, 70%, 80%, 90%, v/v) at 298K were obtained on an FLS 920 combined fluorescence lifetime and steady spectrometer (Japan). The resulting data were processed with Origin Pro v8.0. All media contain minimum DMSO (1% v/v) for better solvency.

**Response to unfolded protein**

The emission intensities and lifetimes of **Re-ERLAD** in solutions containing folded/unfolded β-lactoglobulin (1 mM) at 298K were obtained on an FLS 920 combined fluorescence lifetime and steady spectrometer (Japan). β-lactoglobulin was incubated with **Re-ERLAD** (1 μΜ) in the absence or presence of urea (6 M) for 24 h. The whole process was conducted in a biosafety cabinet. The lifetime at 298 K were obtained on an FLS 920 combined fluorescence lifetime and steady spectrometer (Japan). The resulting data were processed with Origin Pro v8.0. All media contain minimum DMSO (1% v/v) for better solvency.

**Reponse to polarity**

The emisstion intensity and lifetimes of **Re-ERLAD** in mixed solvents containing water and 1,4-dioxane representing different polarities (percentage of 1,4-dioxane: 0%, 20%, 40%, 60%, 80%, 100%, v/v) at 298 K were obtained on an FLS 920 combined fluorescence lifetime and steady spectrometer (Japan). The resulting data were processed with Origin Pro v8.0. All media contain minimum DMSO (1% v/v) for better solvency.

**Response to ions and biomolecules**

The fluorescent lifetime of **Re-ERLAD** in aqueous solution with varies ions and biomolecules (c >1 M) at 298 K were obtained on a FLS 920 combined fluorescence lifetime and steady spectrometer (Japan) after an incubation for a week. The resulting data were processed with Origin Pro v8.0. All media contain minimum DMSO (1% v/v) for better solvency.

**Photosensitization of singlet oxygen (^1^O_2_)**

The capability of **Re-ERLAD** and **py-BODIPY** to photosensitize ^1^O_2_ in PBS, CH_3_CN and CH_2_Cl_2_ at 298K were obtained on a Varian Cary 300 spectrophotometer (USA) with ABDA. The fluorescent quantum yield in PBS were calculated using [Ru(bpy)_3_]Cl_2_ as standards.^[4]^ The resulting data were processed with Origin Pro v8.0. All media contain minimum DMSO (1% v/v) for better solvency.

**Cytotoxicity and photocytoxicity**

The cytotoxicity of **Re-ERLAD** and **py-BODIPY** towards A549 cell lines was determined by MTT assay.

For cytotoxicity in the dark, the compounds were dissolved in DMSO and diluted into gradient concentration with a final DMSO proportion of 1% (v/v). Cells cultured in 96-well plates were grown to confluence before incubated with **Re-ERLAD** and **py-BODIPY** for 44 h. 20 μL of MTT solution (5 mg/mL) was then added to each well. The plates were incubated for an additional 4 h before the media was carefully removed, and DMSO was added (150 μL per well). The plate was shaken for 3 min. The absorbance at 595 nm was measured using a microplate reader (Infinite M200 Pro, Tecan, Switzerland).

For phototoxicity, cells cultured in 96-well plates were grown to confluence before incubated with **Re-ERLAD** and **py-BODIPY** for 20 h. The media containing **Re-ERLAD** or **py-BODIPY** was removed and fresh media without the complexes was added. Then the cells were irradiated with a 450 nm light array (20 mW cm^-2^ ) for 15 min (18 J cm^-2^ ) and further incubated for 24 h. 20 μL of MTT solution (5 mg/mL) was then added to each well. The plates were incubated for an additional 4 h before the media was carefully removed, and DMSO was added (150 μL per well). The plate was shaken for 3 min. The absorbance at 595 nm was measured using a microplate reader (Infinite M200 Pro, Tecan, Switzerland).

**Generation of cellular ROS**

A549 cells were seeded in 35 mm culture dishes (Corning) and incubated for 24 h. The growth media was replaced by PBS solution of **Re-ERLAD** (1 μM) containing DMSO (1%, v/v). The cells were irradiated with a 450 nm light array (20 mW cm^-2^ ) for 15 min (18 J cm^-2^ ) and further incubated for 2 h. The Cell ROX™ Deep Red Reagent was then added into the media and incubated for 30 min before the cells were visualized by confocal microscopy (*λ_ex_* = 633 nm; *λ_em_* = 660 ± 20 nm).

A549 cells were seeded in 6 well plate (Corning) and incubated for 48 h. The growth media was replaced by PBS solution of **Re-ERLAD** (1 μM) containing DMSO (1%, v/v). The cells were irradiated with 450 nm for indicated time and further incubated for 2 h. The Cell ROX™ Deep Red Reagent was then added into the media and incubated for 30 min before the tested by flow cytometry.

**Cellular localization studies**

A549 cells were seeded in 35 mm culture dishes (Corning) and incubated for 24 h. The growth media was replaced by PBS solution of **Re-ERLAD** (1 μM) containing DMSO (1%, v/v) and further incubated for 1 h. The trackers was then added into the media at the indicated concentrations and incubated for 30 min. The media was then removed and the cells were washed with PBS before visualized by confocal microscopy. *λ*_ex_ = 405 nm (**Re-ERLAD**/**py-BODIPY**); 561 nm (ER-Tracker Red); 633 nm (LipidSpot 610); *λ*_em_ = 530 ± 20 nm (**Re-ERLAD**/**py-BODIPY**); 610 ± 20 nm (ER-Tracker Red); 660 ± 20 nm (LipidSpot 610).

**Transmission electron microscopy observations**

A549 cells were seeded in 10 cm culture dishes (Corning) and incubated for 48 h. The growth media was replaced with fresh culture medium with **Re-ERLAD** (1 μM) and DMSO (1%, v/v) and the cells were furhter incubated for 1 h. Then, the cells were irridiated with 450 nm for 10/20 min (20 mW cm^-2^ ) and incubated for 4 h. After that, cells were collected and fixed with 2.5% glutaraldehyde PBS buffer. The cells were then treated with osmium tetroxide, stained with uranyl acetate and lead citrate, and visualized under a transmission electron microscope (JEM 100 CX, JEOL, Tokyo, Japan). Images were photographed using the Eversmart Jazz program (Scitex).

**Western blot of FAM134B**

A549 cells were seeded in 10 cm culture dishes (Corning) and incubated for 48 h. The growth media was replaced with fresh culture medium with **Re-ERLAD** (1 μM) and DMSO (1%, v/v) before further incubated for 1 h. Then, the cells were irridiated with 450 nm light array (20 mW cm^-2^ ) for 15 min (18 J cm^-2^ ) and incubated for 1‒4 h. After that, cells were collected and lysed to extract intracellular proteins (Cell lysis buffer, Beyotime, China)( PMSF, 1 mM). The extracted proteins were quantified with BCA Protein Assay Kit (Beyotime, China), electrophoresed in 12% polyacrylamide gels and transferred to PVDF membranes. The PVDF membranes were blocked with QuickBlock™ (Beyotime, China) and incubated with FAM134B (goat anti rabbit, Abcam, USA) or GAPDH (goat anti rabbit, Abcam, USA) at 4 °C overnight. After washed three times with PBST, the PVDF membranes were incubated with secondary antibody, Goat Anti-Rabbit IgG H&L (HRP) (Abcam, USA), for 2 h at room temperature. Chemiluminescence imaging was carried out by DAB Horseradish Peroxidase Color Development Kit (Beyotime, China) and FluorChem M (Protein Simple, USA).

The positive control was obtained with similar protocol using rapamycin (100 nM, 18 h) or tunicamycin (1 μM, 18 h) as inducers

**Transfection straining of LC3B**

Cells were transfected with RFP-LC3 vector using Lipofectamine 2000 (Life Technologies, USA)^[5]^ and seeded in 35 mm culture dishes (Corning) and incubated for 24 h. The growth media was replaced with fresh culture medium with **Re-ERLAD** (1 μM) and DMSO (1%, v/v) before further incubated for 1 h. Then, the cells were irridiated with 450 nm light array (40 mW cm^-2^ ) for 15 min (36 J cm^-2^ ) and incubated for 1 h. The cells were washed with PBS before visualized by confocal microscopy.

**Immunofluorescent double staining of LC3B and FAM134B**

A549 cells were seeded in 35 mm culture dishes (Corning) and incubated for 24 h. The growth media was replaced with fresh culture medium with **Re-ERLAD** (1 μM) and DMSO (1%, v/v) before further incubated for 1 h. Then, the cells were irridiated with 450 nm for 15 min and incubated for 1 h. After that, cells were fixed with 4% paraformaldehyde, permeabilized with Triton X-100, blocked with QuickBlock™ (Beyotime, China) and incubated with the mixed primary antibody against LC3B (goat anti mouse, Abcam, USA) and FAM134B (goat anti rabbit, Abcam, USA) at 4 °C overnight. After washed three times with PBST, the cells were incubated with the mixed secondary antibody, DyeLight 649-conjugated anti-mouse (Abcam, USA) and DyeLight 561-conjugated anti-rabbit (Abcam, USA), for 2 h at room temperature. Cell imaging was carried out by confocal microscopy. The super-resolution images were collected with Airyscan module of the Carl Zeiss LSM 810. *λ_ex_* = 561 nm (FAM134B); 633 nm (LC3B); *λ_em_* = 610 ± 20 nm (FAM134B); 660 ± 20 nm (LC3B).

**Real-time tracking of ER-buddling and ER-lysosome fusion**

A549 cells were seeded in 35 mm culture dishes (Corning) and incubated for 24 h. The growth media was replaced with fresh culture medium with **Re-ERLAD** (1 μM) and DMSO (1%, v/v) before further incubated for 1 h. ER-Trakcer Red (1 μΜ) or LTDR (200 nM) were then added incubated for 30 min. Cells were irridiated with 450 nm for 15 min before visualized by confocal microscopy. The super-resolution images were collected with Airyscan module of the Carl Zeiss LSM 810. *λ_ex_* = 405 nm (**Re-ERLAD**); 633 nm (LTDR); *λ_em_* = 570 ± 20 nm (**Re-ERLAD**); 660 ± 20 nm (LTDR).

**Real-time tracking of ER turnover via TPFLIM**

A549 cells were seeded in 35 mm culture dishes (Corning) and incubated for 24 h. The growth media was replaced with fresh culture medium with **Re-ERLAD** (1 μM) and DMSO (1%, v/v) before further incubated for 1 h. Cells were then irridiated with a 450 nm laser for 15 min before visualized by TPFLIM. The control experiment was carried out under the same condition except for introducing 3-MA (5 mM) into the culture medium. The lifetime value is given by Becker & Hickl SPCImage. The viscosity is calculated by putting the lifetime into the calibration curve in Fig 1D. *λ_ex_* = 810 nm; 633 nm (LTDR); *λ_em_* = 570 ± 20 nm; 660 ± 20 nm (LTDR).

**Immunofluorescent staining of calreticulin**

A549 cells were seeded in 35 mm culture dishes (Corning) and incubated for 24 h. The growth media was replaced with new culture medium with **Re-ERLAD** (1 μM) and DMSO (1%, v/v) before further incubated for 1 h. 3-MA (5 mM) was added into the culture medium as the positive group.Then, the cells were irridiated with 450 nm for 15 min and incubated for 24 h. After that, cells were fixed with 4% paraformaldehyde and incubated with the primary antibody against calreticulin (goat anti mouse, Abcam, USA) at 4 ℃ overnight. After washed three times with PBST, the cells were incubated with the secondary antibody, DyeLight 649-conjugated anti-mouse (Abcam, USA), for 2 h at room temperature. Cell imaging was carried out by confocal microscopy.

**Supporting Scheme, Figures and Tables**

**Scheme S1**. Synthetic protocols of **py-BODIPY** and **Re-ERLAD**.

**Fig. S1** ESI-MS spectrum of **py-BODIPY** in CH_3_OH.


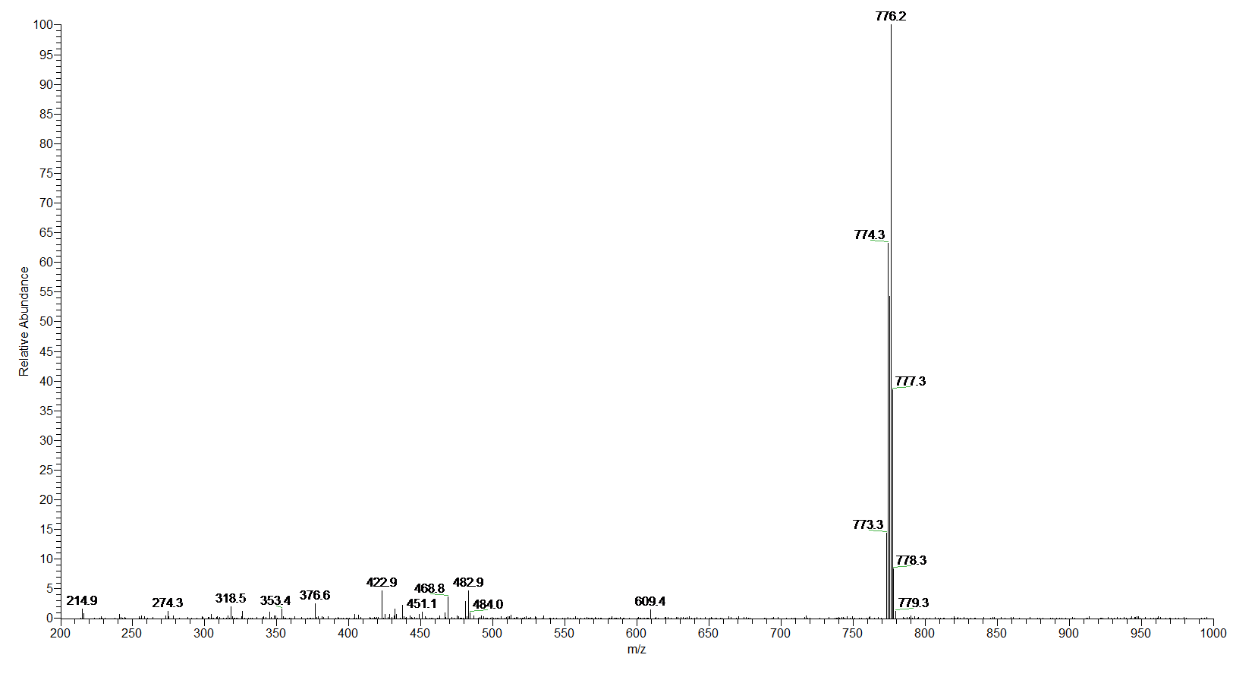


**Fig. S2** ESI-MS spectrum of **Re-ERLAD** in CH_3_OH.





**Fig. S3** ^1^H NMR spectrum of **py-BODIPY** in CD_3_Cl.


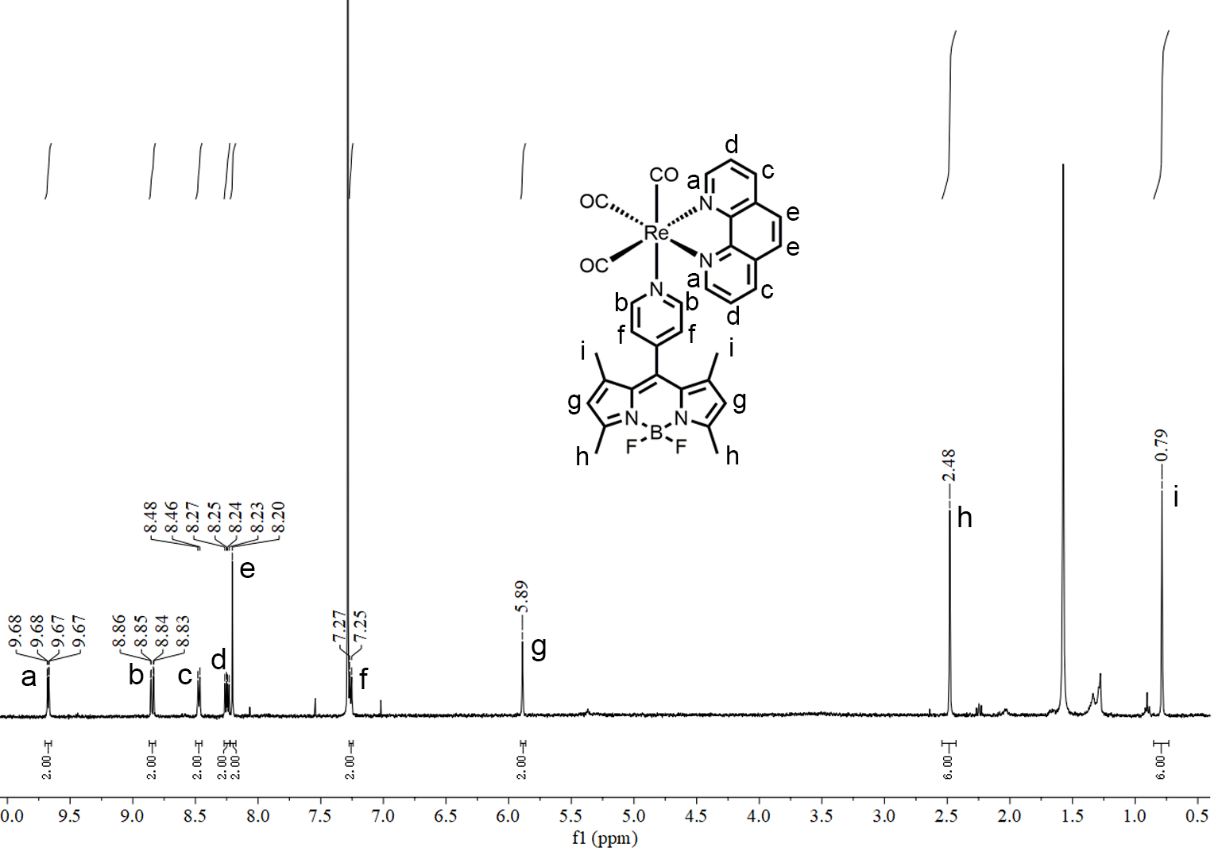


**Fig. S4** ^1^H NMR spectrum of **Re-ERLAD** in CD_3_Cl.


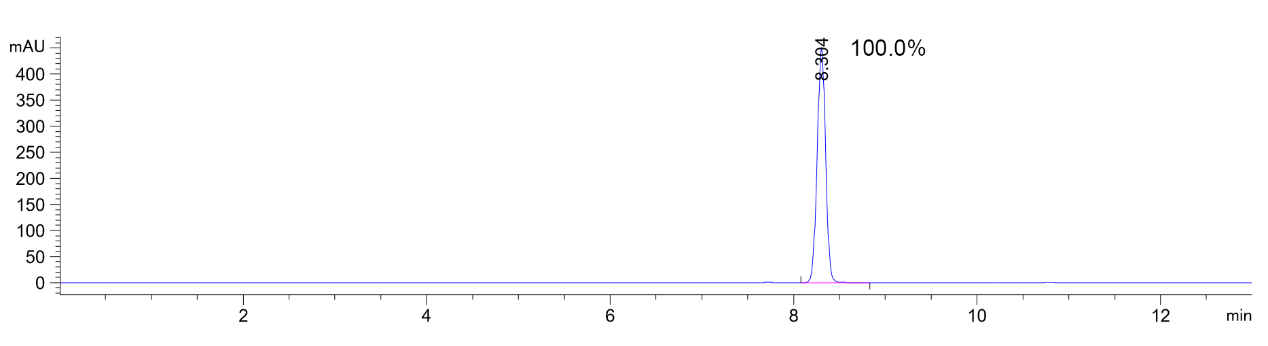


**Fig. S5** HPLC spectrum of **py-BODIPY**.


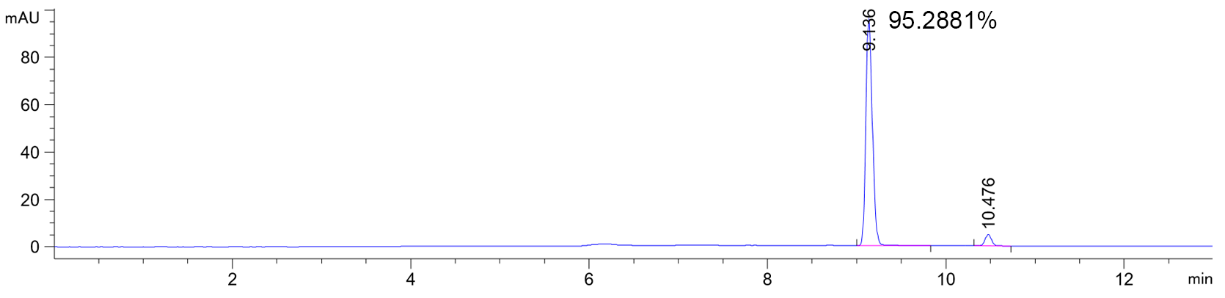


**Fig. S6** HPLC spectrum of **Re-ERLAD**.


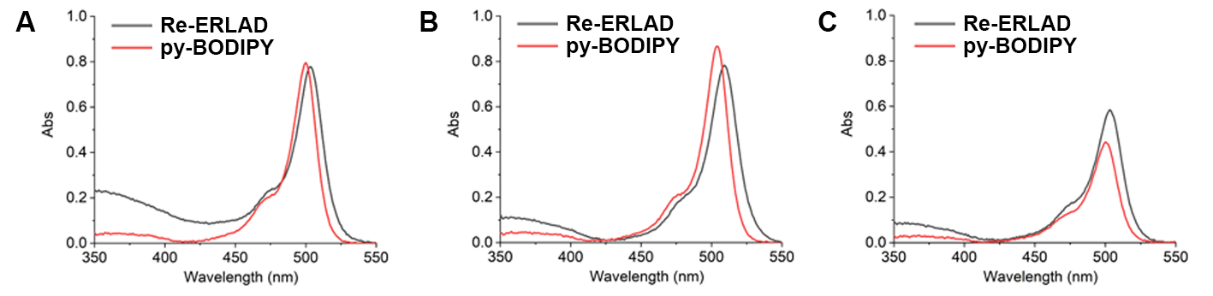


**Fig. S7** UV-Vis spectra of **Re-ERLAD** and **py-BODIPY** in CH_3_CN(A), CH_2_Cl_2_ (B) and PBS (C).


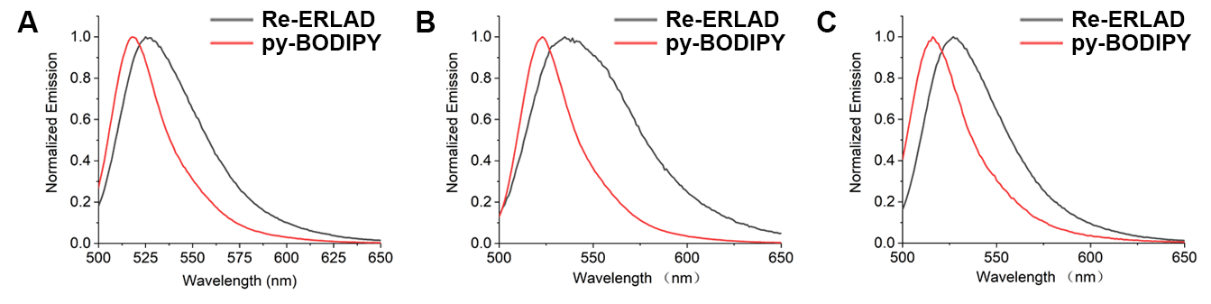


**Fig. S8** Emission spectra of **Re-ERLAD** and **py-BODIPY** in CH_3_CN(A), CH_2_Cl_2_ (B) and PBS (C).


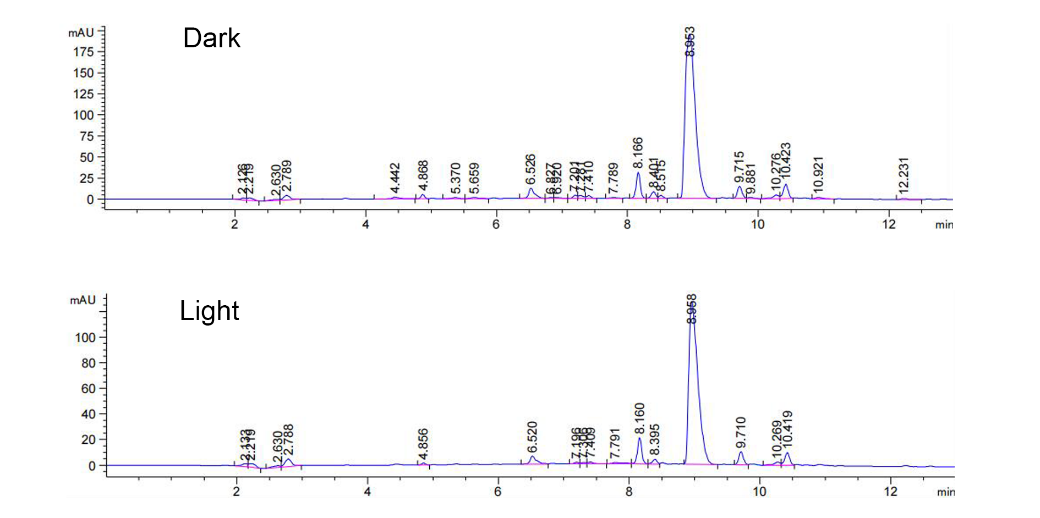


**Fig. S9** HPLC spectra of **Re-ERLAD** in serum. **Re-ERLAD** (100 μM) was dissolved in serum (1% DMSO, v/v), irradiated with 450 nm light array (20 mW cm^-2^ ) for 15 min (18 J cm^-2^ ) and further incubated for 3 days.


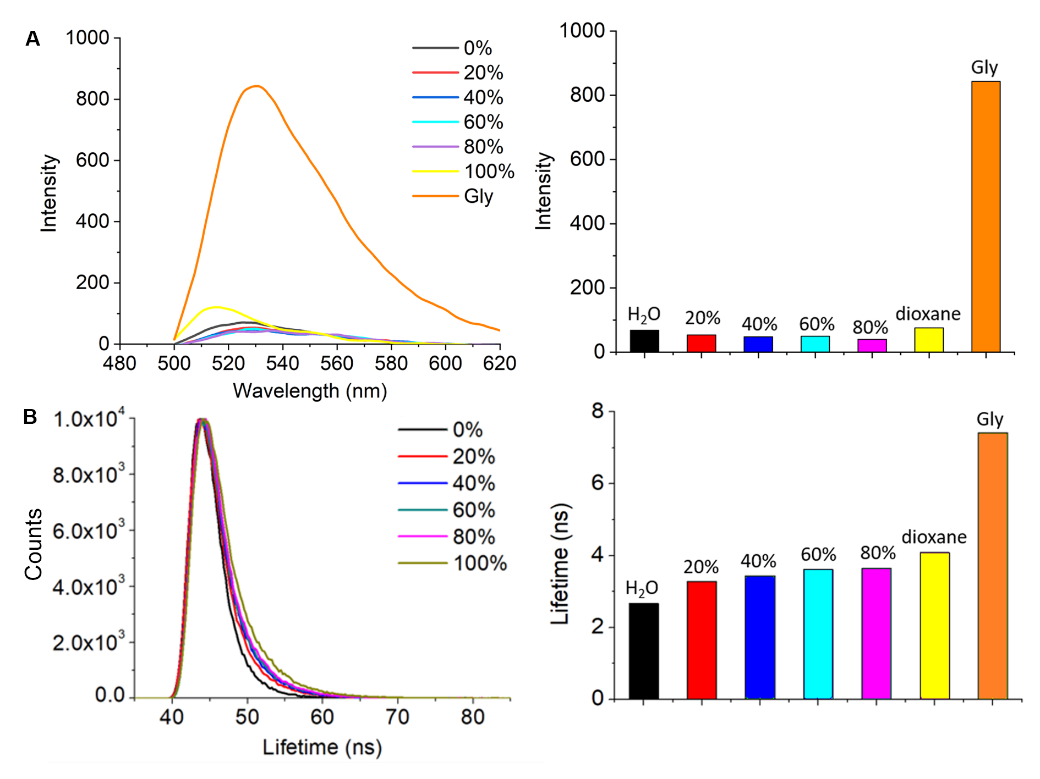


**Fig. S10** Emission intensity (A) and lifetime (B) spectra of **Re-ERLAD** (1 μM) in mixed solvents containing water and 1,4-dioxane representing different polarities (Percentage of 1,4-dioxane: 0%, 20%, 40%, 60%, 80% and 100%). *λ_ex_* **=** 405 nm. Gly: glycerol.


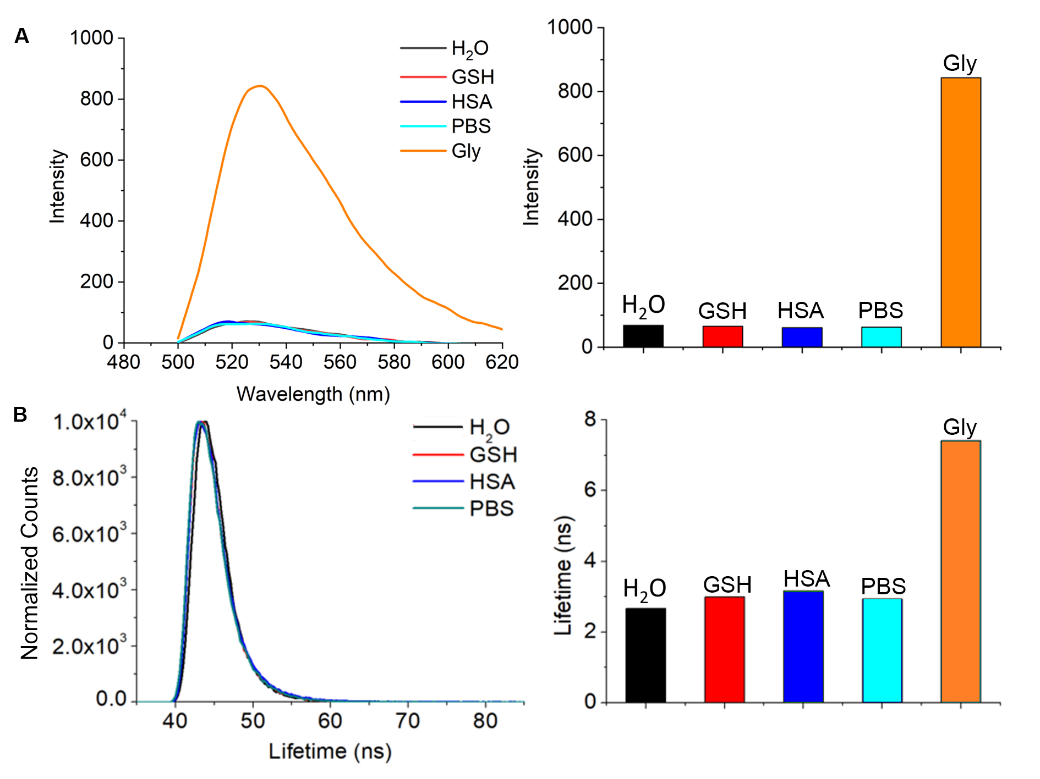


**Fig. S11** Emission intensity (A) and lifetime (B) spectra of **Re-ERLAD** (1 μM) in different solutions: H_2_O, GSH (200 μM) in H_2_O, HSA (200 μM) in H_2_O, PBS (pH = 7.4), Gly-CH_3_OH (Gly/CH_3_OH, v/v = 9:1). *λ_ex_* **=** 405 nm. Gly: glycerol.


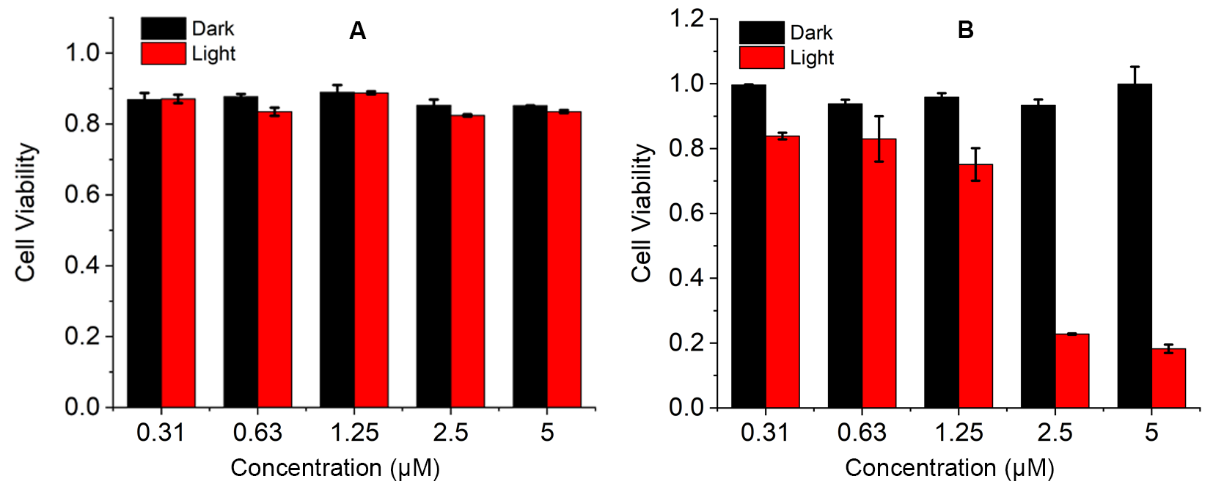


**Fig. S12** Cytotoxicity of **py-BODIPY** (A) and **Re-ERLAD** (B) in the absence and presence of light. Cells were irradiated with 450 nm light array (20 mW cm^-2^ ) for 15 min (18 J cm^-2^ ).

**
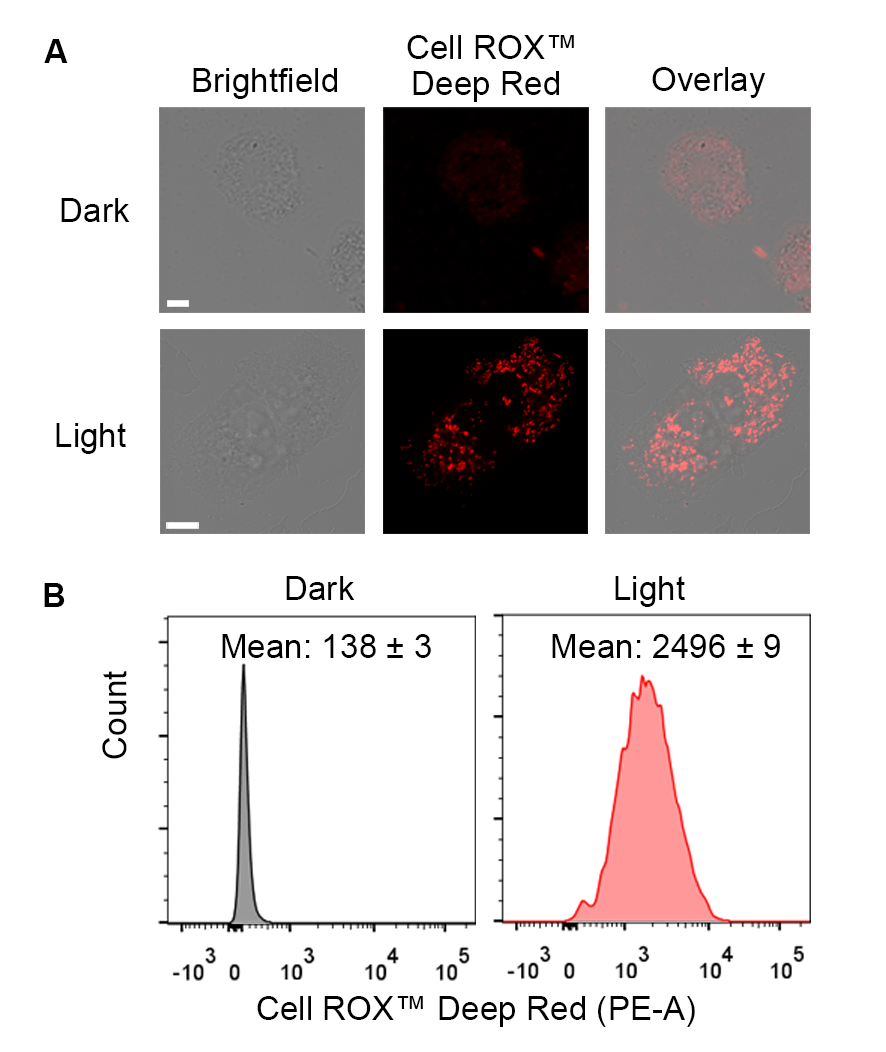
**

**Fig. S13** ROS generation of **Re-ERLAD**. Cells were incubated with **Re-ERLAD** (1 μM）for 1 h, irradiated with 450 nm light array (20 mW cm^-2^ ) for 15 min (18 J cm^-2^ ) stained with Cell ROX™ Deep Red Reagent. The data was collected with (A) confocal microscopy and (B) flow cytometry. *λ*_ex_ **=** 633 nm; *λ*_em_ **=** 660 ± 20 nm. Scale bars: 5 μm.


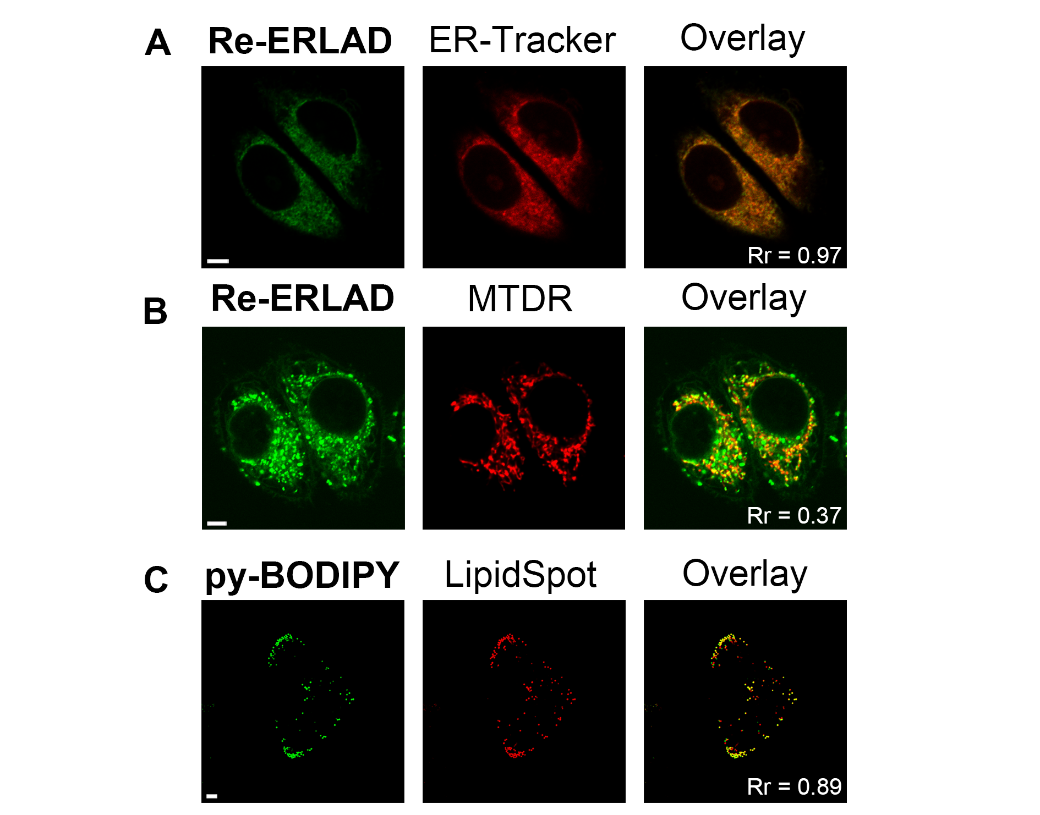


**Fig. S14** Subcellular localization studies of **Re-ERLAD/py-BODIPY** by microscopy. A549 cells were incubated with **Re-ERLAD/py-BODIPY** (1 μM, 1 h) and then stained with (A) MTDR (200 nM, 30 min) (B) ER-Tracker Red (200 nM, 30 min) and (C) LipidSpot 610. *λ*_ex_ **=** 405 nm (**Re-ERLAD/py-BODIPY**); 561 nm (ER-Tracker Red); 633 nm（MTDR/LipidSpot 610); *λ*_em_ **=** 530 ± 20 nm (**Re-ERLAD/py-BODIPY**); 610 ± 20 nm (ER-Tracker Red); 660 ± 20 nm （MTDR/LipidSpot 610）. Overlay: overlay of the 1st and 2nd columns. Scale bars: 5 μm. Rr: Pearson's correlation coefficient.


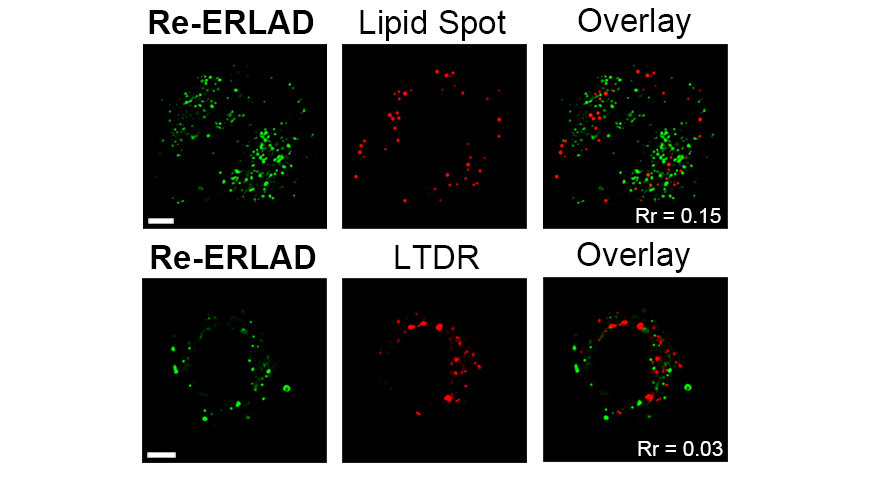


**Fig. S15** Cellular colocalization microscopy image of A549 cells incubated with **Re-ERLAD** (1 μM, 1 h) and LipidSpot 610/LTDR (200 nM, 30 min). Cells were then irradiated with a 450 nm laser for 15 min. *λ*_ex_ **=** 405 nm (**Re-ERLAD**); 633 nm (LipidSpot 610/LTDR); *λ*_em_ **=** 530± 20 nm (**Re-ERLAD**); 660±20 nm (LipidSpot 610/LTDR). Overlay: overlay of the 1st and 2nd columns. Scale bars: 5 μm.

**
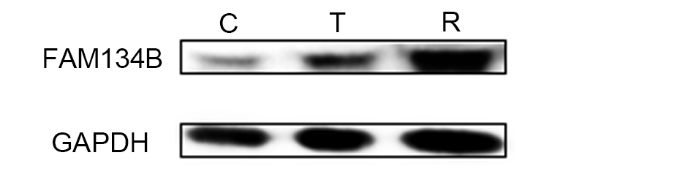
**

**Fig. S16** Western blot of FAM134B. A549 cells were incubated with rapamycin (100 nM, 18 h) or tunicamycin (1 μM, 18 h). C: Control; T: tunicamycin, R: rapamycin.

**

**

**Fig. S17** Cell viability of A549 cells treated with **Re-ERLAD** and 3-MA (5 mM) for 48 h. Cells were irradiated with 450 nm light array (20 mW cm^-2^ ) for 15 min (18 J cm^-2^ ).


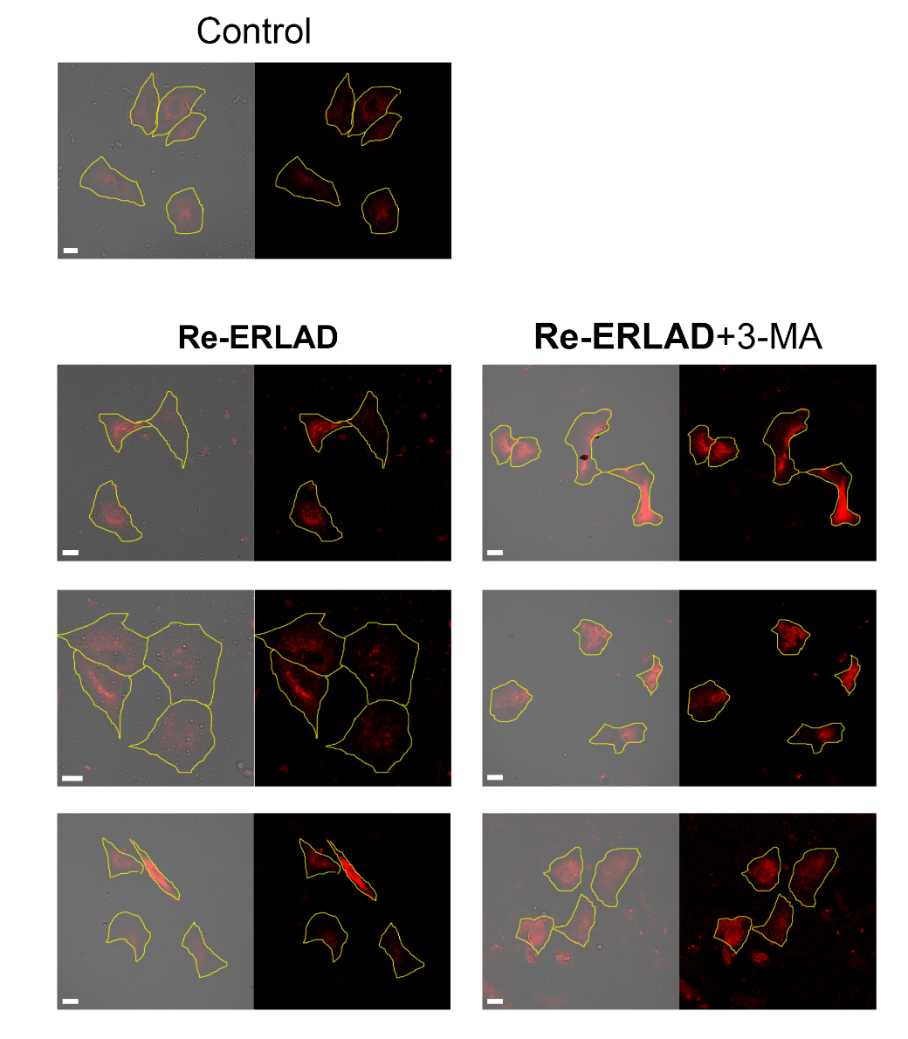


**Fig. S18** Immunofluorescent staining of calreticulin. A549 cells treated with **Re-ERLAD** and 3-MA (5 mM) for 48 h. Cells were irradiated with a 450 nm light array (40 mW cm^-2^ ) for 15 min (36 J cm^-2^ ). Three parallel experiments were carried out for better representation. *λ*_ex_ **=** 633 nm; *λ*_em_ **=** 660 ± 20 nm. Scale bars: 5 μm.

**Table S1**. Photophysical properties of **Re-ERLAD** and **py-BODIPY**

| Compound | Solvent | *λ_em_^a^* | *Ф^b^* | *Ф_Δ_^c^* |
| --- | --- | --- | --- | --- |
| **Re-ERLAD** | PBS | 527 | 0.232 | 0.28 |
|  | CH_3_CN | 525 | 0.136 | / |
|  | CH_2_Cl_2_ | 535 | 0.075 | / |
| **py-BODIPY** | PBS | 516 | 0.281 | 0.07 |
|  | CH_3_CN | 518 | 0.683 | / |
|  | CH_2_Cl_2_ | 523 | 0.837 | / |

*^a^* Maximum wavelengths of one-photon emission spectra (*λ*_em_) in nm. *^b^* Quantum yields of phosphorescence at room temperature were determined using [Ru(bpy)_3_]Cl_2_ in PBS, CH_3_CN and CH_2_Cl_2_. *^c^* Singlet oxygen yield at room temperature using [Ru(bpy)_3_]Cl_2_ as standard.

**References**

[1] Van Houten J and Watts RJ. Temperature dependence of the photophysical and photochemical properties of the tris(2,2'-bipyridyl)ruthenium(II) ion in aqueous solution. *J Am Chem Soc* 1976; **98**: 4853-58;

[2] Tyson DS and Castellano FN. Intramolecular singlet and triplet energy transfer in a ruthenium(II) diimine complex containing multiple pyrenyl chromophores. *J Phys Chem A* 1999; **103**: 10955-60;

[3] Pucci D, Bellusci A and Crispini A et al. Room temperature columnar mesomorphism and high quantum yield phosphorescence in ionic ruthenium(II) 2,2‘-bipyridine-based complexes *J Mat Chem* 2009; **19**: 7643-49.

[4] Wessels JM, Foote CS and Ford WE et al., Photooxidation of tryptophan: O_2_(1Δg) versus electron-transfer pathway. *Photochem Photobiol* 1997; **65**: 96-102.

[5] He L, Liao SY and Tan CP et al. Cyclometalated iridium(III)-β-carboline complexes as potent autophagy-inducing agents. *Chem Commun* 2014; **50**: 5611-14.
